# Trends in Research Related to Ophthalmic OCT Imaging From 2011 to 2020: A Bibliometric Analysis

**DOI:** 10.3389/fmed.2022.820706

**Published:** 2022-04-27

**Authors:** Ziyan Yu, Jie Ye, Fan Lu, Meixiao Shen

**Affiliations:** ^1^School of Ophthalmology and Optometry, Wenzhou Medical University, Wenzhou, China; ^2^Department of Ophthalmology, The Fourth Affiliated Hospital of China Medical University, Shenyang, China; ^3^State Key Laboratory of Optometry, Ophthalmology and Vision Science, Wenzhou, China

**Keywords:** bibliometric analysis, optical coherence tomography (OCT), VOSviewer, ophthalmology (MeSH), OCTA

## Abstract

**Objective:**

The aim of this study was to explore hotspots and global research trends on optical coherence tomography (OCT) in the ophthalmic imaging field using the bibliometric technique.

**Methods:**

Documents related to OCT in the ophthalmic imaging field between 2011 and 2020 were extracted from the Science Citation Index (SCI) Expanded database. Downloaded raw data were analyzed using the VOSviewer and CiteSpace software. Bibliometric networks, including publication number per year, countries, authors, journals, international collaborations, and keywords were constructed.

**Results:**

A total of 4,270 peer-reviewed documents were retrieved, and annual research output in the past 10 years has increased significantly. The largest publishing country was the United States, and the most productive journal was *Investigative Ophthalmology and Visual Science* (*IOVS*). The most active academic institution was the University of California, Los Angeles, and the top rank publishing author was Duker JS. The most co-cited references mainly focused on new emerging OCT techniques such as spectral domain optical coherence tomography (SD-OCT) and optical coherence tomography angiography (OCTA).

**Conclusion:**

The bibliometric analysis of development trends on OCT in the ophthalmic imaging field on various aspects could provide developers or researchers with valuable information to propose future research directions and to pursue further cooperation.

## Introduction

Optical coherence tomography (OCT) was introduced in the early 1990's and has become one of the most successful techniques for displaying the three-dimensional (3D) image of optical tissue biopsy *in vivo* (1). OCT has been applied in clinical research and practice in the ophthalmic field since the 90's. It is a non-invasive, non-contact, and rapid inspection method with a high resolution, and the penetration depth is up to a few millimeters in the eyes (2). OCT can reflect the morphological changes of the retina. It also can measure the thickness and volume of the retina, image the optic nerve disk, and map the ganglion cells in the macular area (3–6). Furthermore, OCT technology can provide 3D structural imaging and functional parameter information of biological tissues. OCT angiography (OCTA) is a non-invasive, new vascular imaging technique that can rapidly image retinal blood vessels, generate high-resolution pictures, and quantify the blood vessel density and blood flow of the retina and choroid *via* novel algorithms. OCTA is important for early diagnosis, follow-up, and development of new preventive and treatment strategies for fundus and optic nerve diseases (7, 8).

Numerous academic documents about OCT in the ophthalmic imaging field have been published since the 1990's. The bibliometric analysis uses statistical and mathematical methods to explore and analyze large volumes of scientific documents. Mapping knowledge domain (MKD) is a method of revealing scientific hotspots and knowledge structures by using document analysis software (VOSviewer and CiteSpace) to create knowledge mapping and categorize published documents (9, 10). In this research, the Science Citation Index (SCI). Expanded database was selected as a primary data source, and the bibliometric analysis to explore trends in ophthalmic OCT research was performed using the tools of MKD. Evaluating research performance in an academic field is crucial to revealing current research hotspots. At present, the knowledge mapping mainly includes keyword co-occurrence analysis (keywords with high-frequency citations) and keyword burst analysis (keywords with the strongest citation bursts) (11).

This bibliometric analysis was designed to analyze the academic output in the ophthalmic OCT field categorically and to visualize its publication trends, including numbers, source journals, author productivity, co-author productivity, co-citation analysis, and international collaborations within the past 10 years. MKD was performed to highlight underexplored areas of OCT research by illustrating the evolution of research in this field.

## Methods

### Database Selection and Search Strategy

The online SCI Expanded database was selected as the data source. “Optical coherence tomography” and “OCT” were set as the search keywords. The language type was limited to “English,” and only “article” published from 2011 to 2020 was considered. We also selected “ophthalmology” as a Web of Science category. Scotland, England, Wales, and Northern Ireland were four administrative regions, and articles from these regions were analyzed separately (not as one country like the United Kingdom). Hong Kong was included under People's Republic of China (PRC). Raw data retrieved from the SCI Expanded database were initially downloaded on January 28, 2021. The file was exported as “plain text,” and “full record and cited references” was selected. Basic information of each document, such as countries, organizations, authors, title, journal, abstract, keywords, and references, were extracted.

### Mapping Analysis

In this research, a visualized bibliometric analysis was generated using the VOSviewer software (www.vosviewer.com) version 1.6.13 (10). This software is used to construct visualized bibliometric maps and generate node-link maps, including research trends information, such as countries, publications, and researchers, and the network information of the co-cited reference analysis and co-authorship analysis. Keywords are used to express the theme of the scientific literature, and the clustering of similar keywords resulted in co-occurrence keyword clusters, which could be used to explore the knowledge structure and hotspots in this research field. The CiteSpace 5.6.R2 software (Drexel University, Philadelphia, PA) was used to capture keywords with a strong burst, which could be considered as predictors of research frontiers.

## Results

### Annual Distributions of Publications

**Figure 1:**
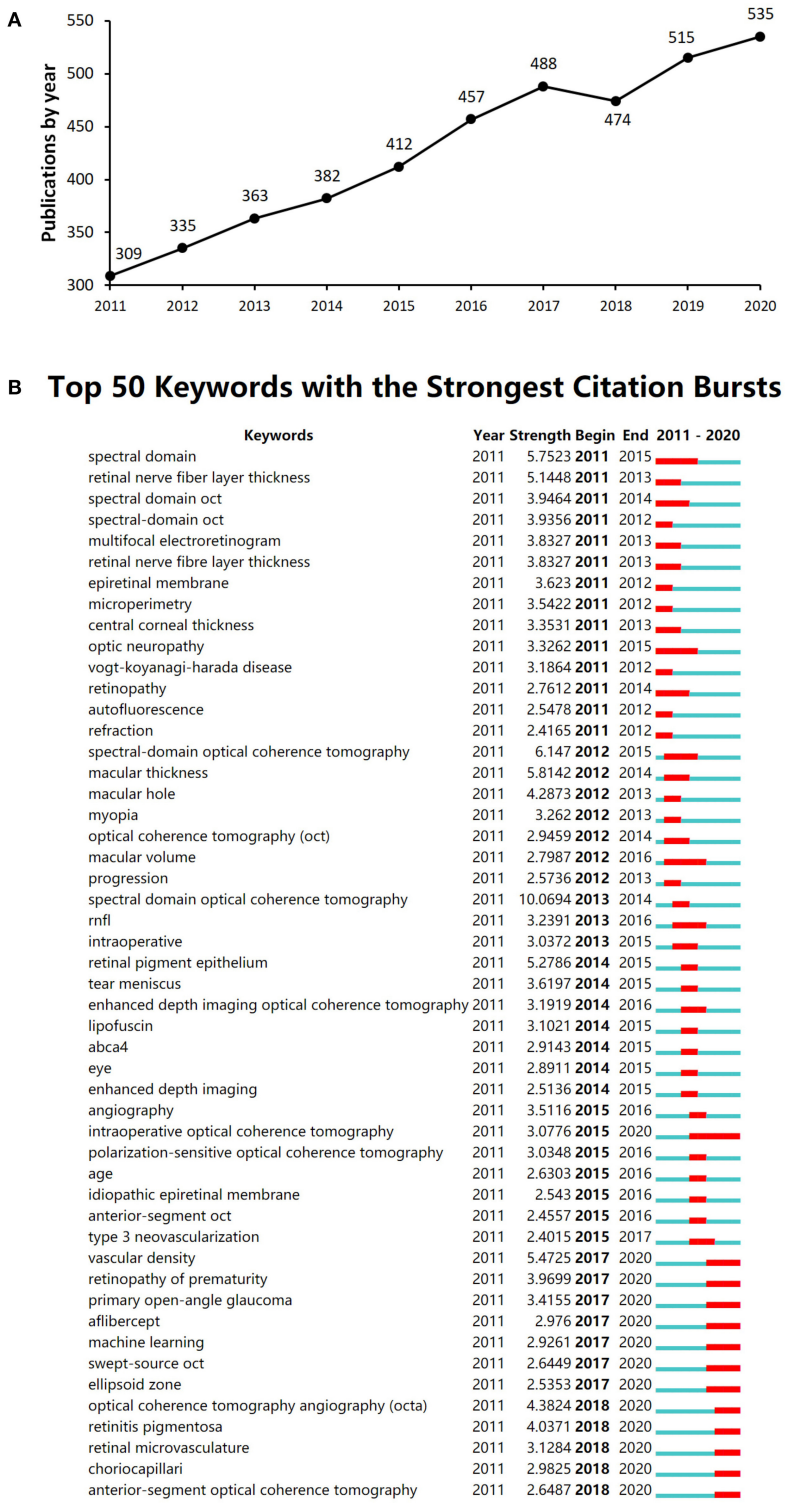
**(A)** The annual publication number from 2011 to 2020 in the ophthalmic optical coherence tomography (OCT) field. **(B)** The top 50 burst keywords from 2011 to 2020 in the ophthalmic OCT field.

Derived from the selection criteria, 4,270 documents were retrieved from the SCI Expanded database related to ophthalmic OCT imaging from 2011 to 2020. The publication number rose gradually from 309 in 2011 to 535 in 2020 (Figure 1A). The top 50 strong citation burst keywords were extracted. Among these keywords, “spectral domain,” “spectral domain OCT,” and “spectral domain Optical Coherence Tomography” showed citation bursts from 2011 to 2015, in line with the rise of published documents (Figure 1B).

### Top Ten Productive Countries

**Figure 2:**
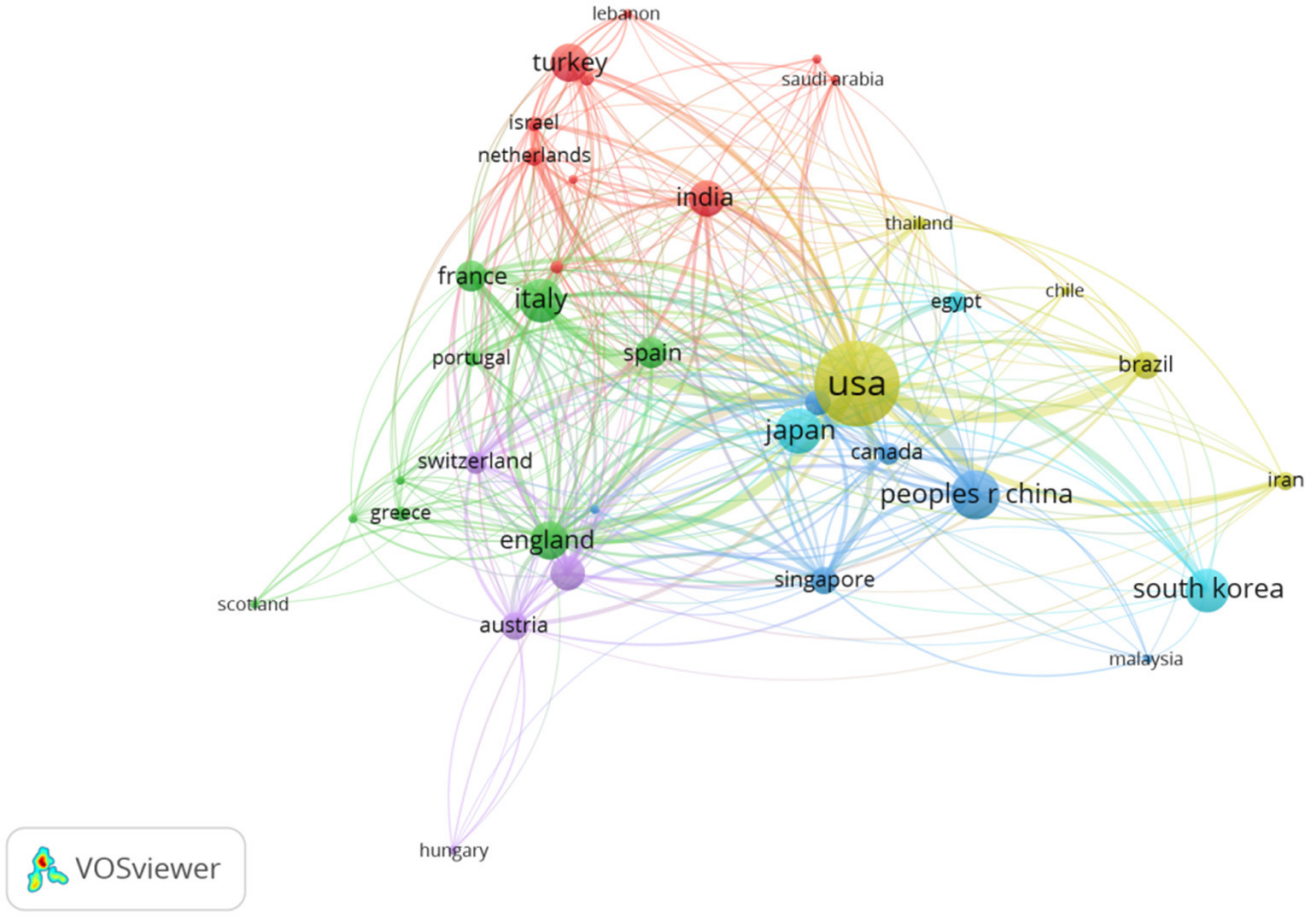
Distribution of main countries from 2011 to 2020 in the ophthalmic OCT field. The minimum number of a country's number was set as 10. A total of 38 countries met the threshold of 71 countries.

A total of 4,270 documents originated from 71 countries. The top 10 countries accounted for 97% (4,141) of all documents in the OCT ophthalmic field (Table 1). The United States ranked first (1,371, 32.1%), China (462, 10.8%) ranked second, and Japan (401, 9.4%) ranked third among the top 10 countries. In the citation analysis, the United States (37,578 citations) took the first place, followed by Japan (7,624 citations) and China (7,263 citations). Communication degree among countries was reflected by the country co-authorship analysis. Nodes represent the influence of each country; the larger the node, the greater the influence. Links between nodes revealed the cooperation degree of countries; the distance and strength of links correspond to the closeness of cooperation in the OCT field (Figure 2).

**Table 1:** Top ten productive countries from 2011 to 2020 in the ophthalmic optical coherence tomography (OCT) field.

**Rank**	**Country/District**	**Count (%)**	**Citations**
1	USA	1,371 (32.1)	37,578
2	China	462 (10.8)	7,263
3	Japan	401 (9.4)	7,624
4	Italy	355 (8.3)	6,106
5	South Korea	350 (8.2)	6,029
6	England	267 (6.3)	5,153
7	Turkey	267 (6.3)	1,951
8	India	256 (6.0)	2,851
9	Germany	222 (5.2)	4,657
10	France	190 (4.4)	4,340

*The percentages were calculated by dividing the number of row count by the total number of publications (n = 4,270)*.

### Top Ten Organizations

**Figure 3:**
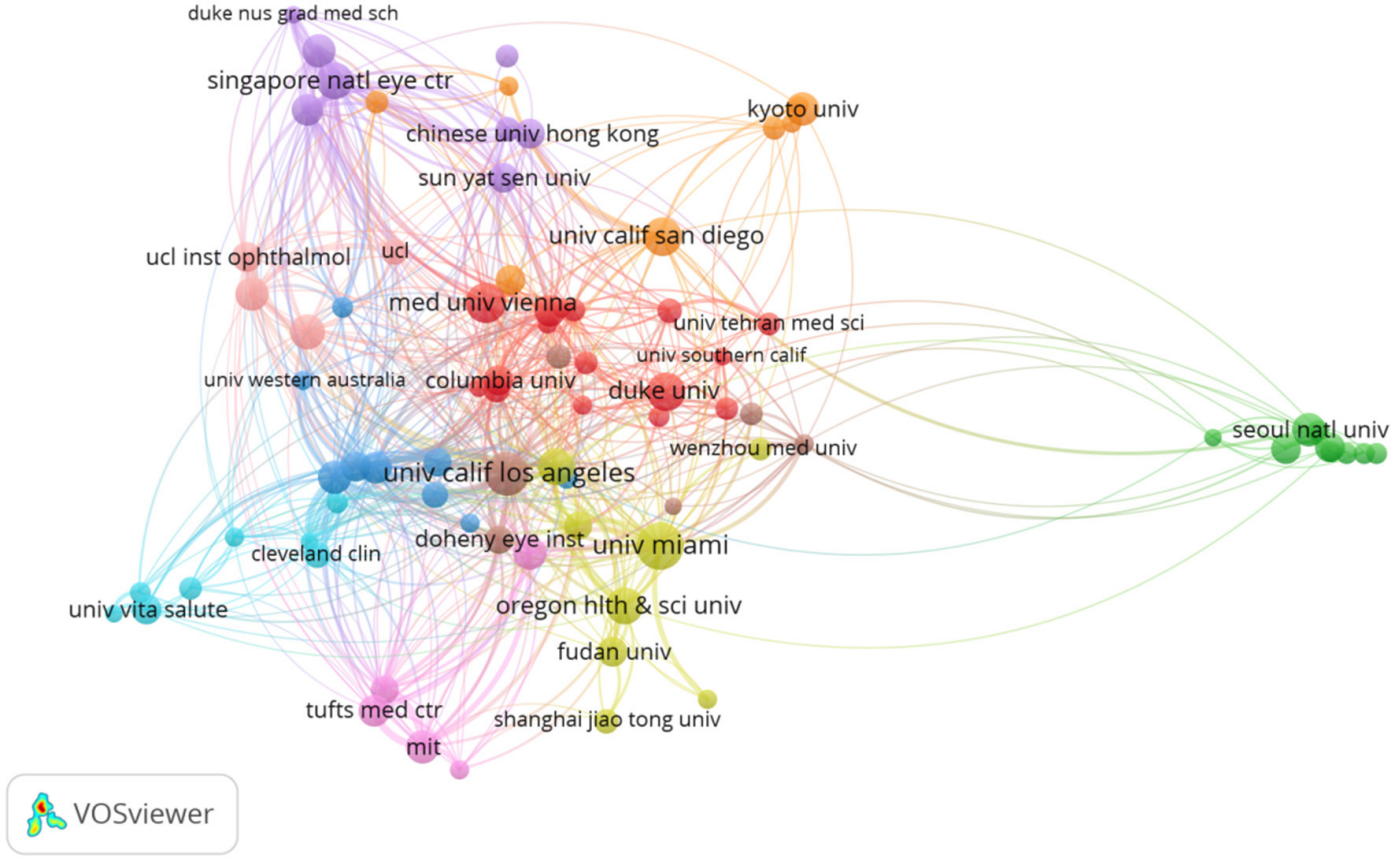
Cooperation network of main research organizations from 2011 to 2020 in the ophthalmic OCT field. The minimum number of documents of an organization was set as 20. A total of 83 organizations of the 2,761 organizations met the threshold.

A total of 4,270 documents were obtained from 2,761 organizations, and the top 10 organizations accounted for 20.7% (885 documents) (Table 2). Using the co-authorship analysis, MKD of research organizations' distribution was constructed (Figure 3). The node size represented the number of published documents, and the link strength shows the closeness of cooperation.

**Table 2:** Top ten productive organizations from 2011 to 2020 in the ophthalmic OCT field.

**Rank**	**Organization**	**Country**	**Documents (%)**	**Citations**
1	University of California Los Angeles	USA	126 (3.0)	3,010
2	University of Miami	USA	121 (2.8)	3,677
3	Medical University of Vienna	Austria	98 (2.3)	1,998
4	Duke University	USA	89 (2.1)	1,557
5	Oregon Health and Science University	USA	84 (2.0)	3,536
6	Singapore National Eye Center	Singapore	82 (1.9)	1,610
7	University of California San Diego	USA	81 (1.9)	2,882
8	Moorfields Eye Hospital	England	69 (1.6)	1,823
9	Moorfields Eye Hospital NHS Foundation Trust	England	69 (1.6)	1,340
10	National University of Singapore	Singapore	66 (1.5)	1,361

*The percentages were calculated by dividing the number of row count by the total number of (n = 4,270)*.

### Distribution of Authors and Co-authors

**Figure 4:**
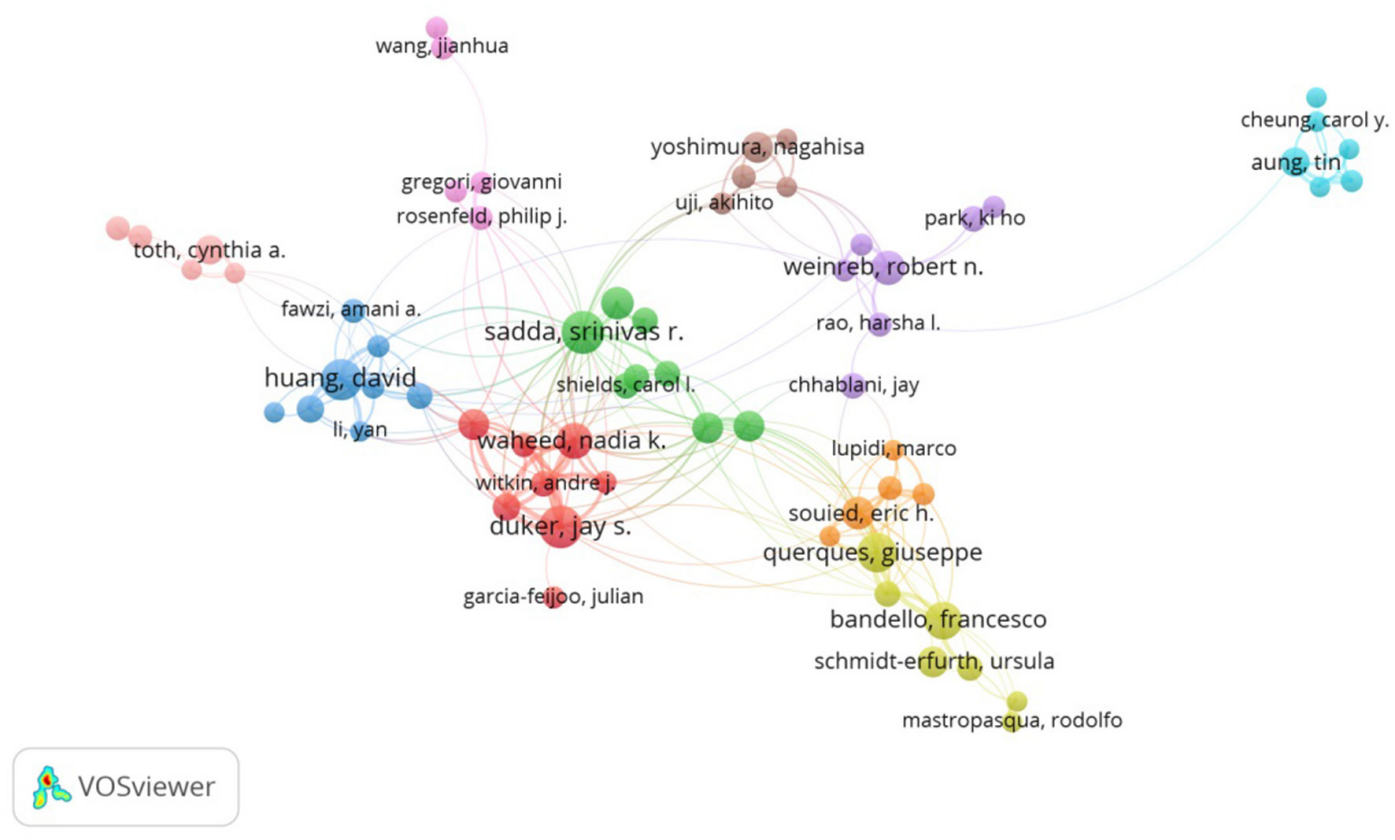
Co-authorship network of productive authors from 2011 to 2020 in the ophthalmic OCT field. The minimum number of documents of an author was set as 20. A total of 68 authors met the threshold of the 12,960 authors that were involved in this study.

Approximately 12,960 authors contributed to 4,270 documents. Among these authors, Duker JS (86 articles) contributed the most, followed by Sadda SR (85 articles) and Huang D (80 articles). Author co-citations were analyzed to reveal authors' relative influence in the OCT field. Spaide RF (1,620 co-citations), followed by Jia YL (846 co-citations) and Leung CKS (695 co-citations) were the three top-ranked authors (Table 3). The co-authorship analysis revealed the MKD for distribution of research teams (Figure 4). The size of the node represented the number of documents. The greater links meant the higher density cooperation between these authors.

**Table 3:** Top ten productive authors and co-cited authors from 2011 to 2020 in the ophthalmic OCT field.

**Rank**	**Author**	**Count**	**Co-cited author**	**Citations**
1	Duker JS	86	Spaide RF	1,620
2	Sadda SR	85	Jia YL	846
3	Huang D	80	Leung CKS	695
4	Querques G	68	Quigley HA	548
5	Bandello F	64	Huang D	508
6	Waheed NK	58	Hood DC	422
7	Weinreb RN	55	Mwanza JC	376
8	Souied EH	50	Medeiros FA	349
9	Keane PA	47	Jonas JB	349
10	Sarraf D	46	Ehlers JP	318

### Distribution of Journals

A total of 4,270 academic documents originated from 66 journals. The top 10 journals are shown in Table 4. The top three journals, *Investigative Ophthalmology and Visual Science* (512, 12.0%), *Retina-The Journal of Retinal and Vitreous Diseases* (437, 10.2%), and *American Journal of Ophthalmology* (305, 7.1%), accounted for 29.4% of all documents.

**Table 4:** Top ten main source journals from 2011 to 2020 in the ophthalmic OCT field.

**Rank**	**Journal**	**Country**	**Count**	**% of 4,270**
1	Investigative Ophthalmology and Visual Science	United States	512	12.0
2	Retina-the Journal of Retinal and Vitreous Diseases	United States	437	10.2
3	American Journal of Ophthalmology	United States	305	7.1
4	British Journal of Ophthalmology	England	224	5.2
5	Graefes Archive for Clinical and Experimental Ophthalmology	United States	218	5.1
6	Journal of Glaucoma	United States	168	3.9
7	Ophthalmology	United States	165	3.9
8	Ophthalmic Surgery Lasers and Imaging Retina	United States	159	3.7
9	Acta Ophthalmologica	United States	140	3.3
10	Journal of Ophthalmology	England	125	2.9

### Distribution of Co-cited References

A total of 44,080 cited references were retrieved in the co-citation analysis. Moreover, 50 was set as the minimum citation number of a cited reference. The top 10 co-cited references are shown in Table 5.

**Table 5:** Top ten co-cited references in OCT research from 2011 to 2020 in the ophthalmic OCT field.

**Rank**	**Co-cited reference**	**Title**	**Citations**
1	Huang D. Science. 1991;254:1178	Optical coherence tomography.	407
2	Jia YL. Opt Express. 2012;20:4710	Split-spectrum amplitude-decorrelation angiography with optical coherence tomography.	344
3	Spaide RF. Jama Ophthalmol. 2015;133:45	Retinal vascular layers imaged by fluorescein angiography and optical coherence tomography angiography.	324
4	Spaide RF. Am J Ophthalmol. 2008;146:496	Enhanced depth imaging spectral-domain optical coherence tomography	284
5	Spaide RF. Retina-J Ret Vit Dis. 2015;35:2163	Image artifacts in optical coherence tomography angiography.	193
6	Margolis R. Am J Ophthalmol. 2009; 147:811	A pilot study of enhanced depth imaging optical coherence tomography of the choroid in normal eyes.	171
7	Jia YL. Ophthalmology. 2014;121:1435	Quantitative optical coherence tomography angiography of choroidal neovascularization in age-related macular degeneration.	165
8	Jia YL. Ophthalmology. 2014;121:1322	Optical coherence tomography angiography of optic disc perfusion in glaucoma.	153
9	Bland JM. Lancet. 1986;1:307	Statistical methods assessing agreement between two methods clinical measurement.	150
10	Fujiwara T. Am J Ophthalmol. 2009;148:445	Enhanced depth imaging optical coherence tomography of the choroid in highly myopic eyes.	130

### Distribution of Key Words: Hotspots of OCT Study

Through the co-occurrence analysis of high-frequency keywords, the research hotspots of OCT were identified. The minimum co-occurrence number of a keyword was set as 20. Among the extracted 7,293 keywords that were involved in OCT, 116 keywords met the threshold. Based on the network, the keywords with similarities were clustered. The top 10 keywords for each cluster are listed in Table 6.

**Table 6:** Co-occurrence analysis of keywords. Top 10 keywords in the 3 clusters.

**Cluster 1**	**Cluster 2**	**Cluster 3**
Optical coherence tomography (OCT) (1,121)	Age-related macular degeneration (103)	Glaucoma (253)
Optical coherence tomography angiography (OCTA) (397)	Diabetic retinopathy (90)	Retinal nerve fiber layer (139)
Spectral domain optical coherence tomography (SD-OCT) (260)	Choroidal neovascularization (69)	Anterior segment optical coherence tomography (AS-OCT)(118)
Retina (115)	Diabetic macular edema (48)	Optic nerve head (49)
Choroidal thickness (99)	Macular edema (48)	Retinal nerve fiber layer thickness (40)
Imaging (82)	Macular hole (47)	Cornea (33)
Fluorescein angiography (78)	Vitrectomy (40)	Trabeculectomy (31)
Foveal avascular zone (58)	Myopia (40)	Optic nerve (30)
Enhanced depth imaging optical coherence tomography (EDI-OCT) (53)	Uveitis (39)	Keratoconus (25)
Vessel density (51)	Central serous chorioretinopathy (31)	Anterior chamber angle (23)

*The numbers in brackets represent the frequency of keywords according to the co-occurrence analysis*.

## Discussion

In the present analysis, 4,270 documents related to ophthalmic OCT imaging from 2011 to 2020 were identified through the SCI Expanded database. As an important research index, the amount of academic documents is an important research index and can indicate the development directions in a research field. The annual publication number rose steadily in the past 10 years, representing the rapid development of OCT in the ophthalmic field. The University of California, Los Angeles, the University of Miami, and the Medical University of Vienna in Australia were the most productive and the most active institutions in international collaborations. The co-authorship and author co-citations analysis could provide information regarding author's contribution and relative influence guiding researchers and scientists to pursue scientific cooperation in the OCT imaging field. The United States, China, and Japan were the leading countries and made a great contribution to the publication in the ophthalmic OCT field (12–20). The United States is the key node cooperating with China, England, Italy, and other countries. Geographical distance may not be an influential factor affecting international cooperation. The co-citation analysis revealed related topics in high-quality academic documents. The top 10 co-cited references were mainly pertained to new technique, which were regarded as milestones in the history of OCT development. Notably, a publication regarding statistical methods used for accessing the degree of agreement ranked top 9 (19).

The keyword co-occurrence analysis and strongest burst keywords represented the evolution trends of research hotspots in this field and were considered to reflect the search theme. Choriocapillari and retinal microvasculature were the latest burst keywords, indicating that new emerging vascular imaging techniques such as *en face* OCT might be potential research hotspots in the future (21). “Machine learning” is the burst keyword from 2017 to 2020. Artificial intelligence (AI)-based algorithms can enhance quality and efficiency and has been widely used in medical imaging at present (22, 23). Keyword co-occurrence cluster analyses showed that the frontier discipline and internal structure related to the ophthalmic OCT literature mainly formed three clusters (Table 6), and each cluster was summarized in a specific theme. Cluster 1 is linked with imaging technique development [i.e., spectral-domain OCT (SD-OCT), OCTA, swept-source OCT (SS-OCT), and enhanced depth imaging OCT (EDI-OCT)] (24–28). Cluster 2 is linked with retinal and choroidal diseases (e.g., age-related macular degeneration, diabetic retinopathy, high myopia, and uveitis) (29–31). Cluster 3 is linked with glaucoma and cornea diseases (e.g., glaucoma, retinal nerve fiber layer, anterior segment OCT (AS-OCT), and keratoconus) (32, 33). These research hotspots mainly focused on the mechanism, pathology, biological measurement, diagnosis, and treatment guidance of ocular diseases.

However, some methodological limitations may exist in the present analysis. The result only included the perspective of application in the ophthalmic field, although OCT and OCTA are now used extensively in the neurological research field. In addition, the language was restricted to English, and linguistic bias may exist. The SCI was selected in our study, and PubMed, Google Scholar, ProQuest, PsycINFO, and other databases were not included. The SCI Expanded database is enough for bibliometric analysis, but there is minimal difference between SCI Expanded and WoSCC for the retrieved documents' number. Furthermore, some bibliometric experts use “front page” as a filter to improve the bibliometric analysis and to reduce unrelated documents for analysis (34).

## Conclusion

This study reviewed academic publications for the past decade in the ophthalmic OCT imaging field to provide a global view of the current research output. The valuable information and guidance provided by the current study are crucial for global ophthalmologists and OCT developers to propose future research directions and to seek collaboration opportunities in the ophthalmic OCT imaging field.

## Author Contributions

FL and MS: design of this study and supervision. ZY and JY: literature search and data analysis. ZY, JY, FL, and MS: manuscript writing and editing. All authors approved the final version of the article.

## Funding

This study was supported by the Natural Science Foundation of China (NSFC 82000877) and the National Key Project of Research and Development Program of Zhejiang Province (2019C03045). The sponsors did not participate in the design or implementation of this study.

## Conflict of Interest

The authors declare that the research was conducted in the absence of any commercial or financial relationships that could be construed as a potential conflict of interest.

## Publisher's Note

All claims expressed in this article are solely those of the authors and do not necessarily represent those of their affiliated organizations, or those of the publisher, the editors and the reviewers. Any product that may be evaluated in this article, or claim that may be made by its manufacturer, is not guaranteed or endorsed by the publisher.
